# Morphological and molecular characterization of *Contracaecum australe* (Nematoda: Anisakidae) parasitizing *Phalacrocorax brasilianus* (Aves: Phalacrocoracidae) on the north coast of Brazil

**DOI:** 10.1590/S1984-29612023039

**Published:** 2023-06-30

**Authors:** Ricardo Luis Sousa Santana, Elaine Lopes de Carvalho, José Ledamir Sindeaux, Michele Velasco Oliveira da Silva, Raul Henrique da Silva Pinheiro, Evonnildo Costa Gonçalves, Elane Guerreiro Giese

**Affiliations:** 1 Laboratório de Histologia e Embriologia Animal, Instituto de Saúde e Produção Animal - Universidade Federal Rural da Amazônia - UFRA, Belém, PA, Brasil; 2 Programa de Pós-graduação em Saúde e Produção Animal na Amazônia, Instituto de Saúde e Produção Animal, Universidade Federal Rural da Amazônia - UFRA, Belém, PA, Brasil; 3 Laboratório de Sanidade em Organismos Aquáticos, Universidade Federal Rural da Amazônia - UFRA, Belém, PA, Brasil; 4 Laboratório de Tecnologia Biomolecular, Universidade Federal do Pará - UFPA, Belém, PA, Brasil

**Keywords:** Parasites, birds, scanning electron microscopy, nuclear ribosomal DNA, internal transcribed spacers (ITS), Parasitos, aves, microscopia eletrônica de varredura, DNA ribossomal nuclear, espaçadores transcritos internos (ITS)

## Abstract

For the first time in Brazil, *Contracaecum australe* is recorded parasitizing *Phalacrocorax brasilianus* (Aves, Suliformes, Phalacrocoracidae) from the Marine Extractive Reserve of Soure on Marajó Island, Brazilian Amazon. Its morphology revealed a body with a transversally striated cuticle, smooth or slightly cleft interlabia, lips with auricles, labial papillae, and conspicuous amphids. In males, the presence of the median papilla on the upper lip of the cloaca and spicules that reach almost half of the body of the parasite. These morphological characters, added to the number and distribution of the pre- and postcloacal papillae of the male specimens, and supported by the molecular phylogeny from the analysis of the ITS-1, 5.8S and ITS-2 genes, allowed the identification of these parasites.

## Resumo

Pela primeira vez no Brasil, *Contracaecum australe* é registrado parasitando *Phalacrocorax brasilianus* (Aves, Suliformes, Phalacrocoracidae) da Reserva Extrativista Marinha de Soure na Ilha de Marajó, Amazônia brasileira. Sua morfologia revelou corpo com cutícula estriada transversalmente, interlábios lisos ou levemente fendidos, lábios com aurículas, papilas labiais e anfídeos conspícuos. Nos machos, observa-se a presença da papila mediana no lábio superior da cloaca e espículos que atingem quase a metade do corpo do parasito. Esses caracteres morfológicos, somados ao número e distribuição das papilas pré e pós-cloacais dos espécimes machos, e apoiados pela filogenia molecular a partir da análise dos genes ITS-1, 5.8S e ITS-2, permitiram a identificação desses parasitos.

## Introduction

Nematodes of the Anisakidae family infect a variety of aquatic organisms at various developmental stages of their life cycle (Anderson, 2000). Among the anisakids, the genus *Contracaecum* Railliet & Henry, 1912, stands out, having been recorded in several locations on the planet (Biolé et al., 2012). They are parasites described in freshwater, brackish, and marine ecosystems that use fish as intermediate and/or paratenic hosts and aquatic mammals and piscivorous birds as definitive hosts (Anderson, 2000; Mattiucci et al., 2008; Mattiucci & Nascetti, 2008; Shamsi et al., 2009a; Garbin et al., 2011; Shamsi, 2014).

For the South American continent, six species of *Contracaecum* parasitizing *Phalacrocorax brasilianus* (Gmelin, 1789) are recorded: *C*. *caballeroi* Bravo-Hollis, 1939; *C*. *travassosi* Lent & Freitas, 1948; *C*. *rudolphii* Hartwich, 1964 (Syn. *C*. *spiculigerum*); *C*. *multipapillatum* (Drasche, 1882) Baylis, 1920; *C*. *australe* Garbin, Mattiucci, Paoletti, González-Acuña and Nascetti, 2011; *C*. *jorgei* Sardella, Mancini, Salinas, Simões and Luque, 2020 (Torres et al., 2000; Amato et al., 2006; Violante-González et al., 2011, 2015; Garbin et al., 2011; Biolé et al., 2012; González-Acuña et al., 2020; Sardella, et al., 2020).

In terms of Brazilian avifauna, eight species of *Contracaecum* parasitizing different species of birds have been recorded to date: *C*. *microcephalum* (Rudolphi, 1819) Baylis, 1920; *C*. *multipapillatum*; *C*. *granulosum* (Schneider, 1866) Baylis, 1932; *C*. *crenulatum* Schuurmans-Stekhoven, 1937; *C*. *caballeroi*; *C*. *pelagicum* Johnston & Mawson, 1942; *C*. *plagiaticium* Lent & Freitas, 1948; and *C*. *rudolphii* (Vicente et al., 1995; 1996; Amato et al., 2006), but only *C*. *rudolphii* has been recorded parasitizing the Neotropical cormorant in Brazilian territory (Vicente et al., 1996; Amato et al., 2006).

The species *C*. *australe* was described for the first time in lagoon Santa Elena in Chile, as a parasite of *P*. *brasilianus*, using morphological and molecular analyses (Garbin et al., 2011). Biolé et al. (2012), recorded the species on the same host in central Argentina and later *P*. *gaimardi* Lesson & Garnot, 1828 was added as a new host for *C*. *australe*, the southernmost record of the species in Argentina, thus expanding its geographical distribution and definitive host range (Garbin et al., 2014).

Given the above, this study aims to investigate the nematode parasites of *Phalacrocorax brasilianus* from the Marine Extractive Reserve of Soure, Marajó Island, Pará, employing the perspective of integrative taxonomy.

## Material and Methods

**Figure 1:**
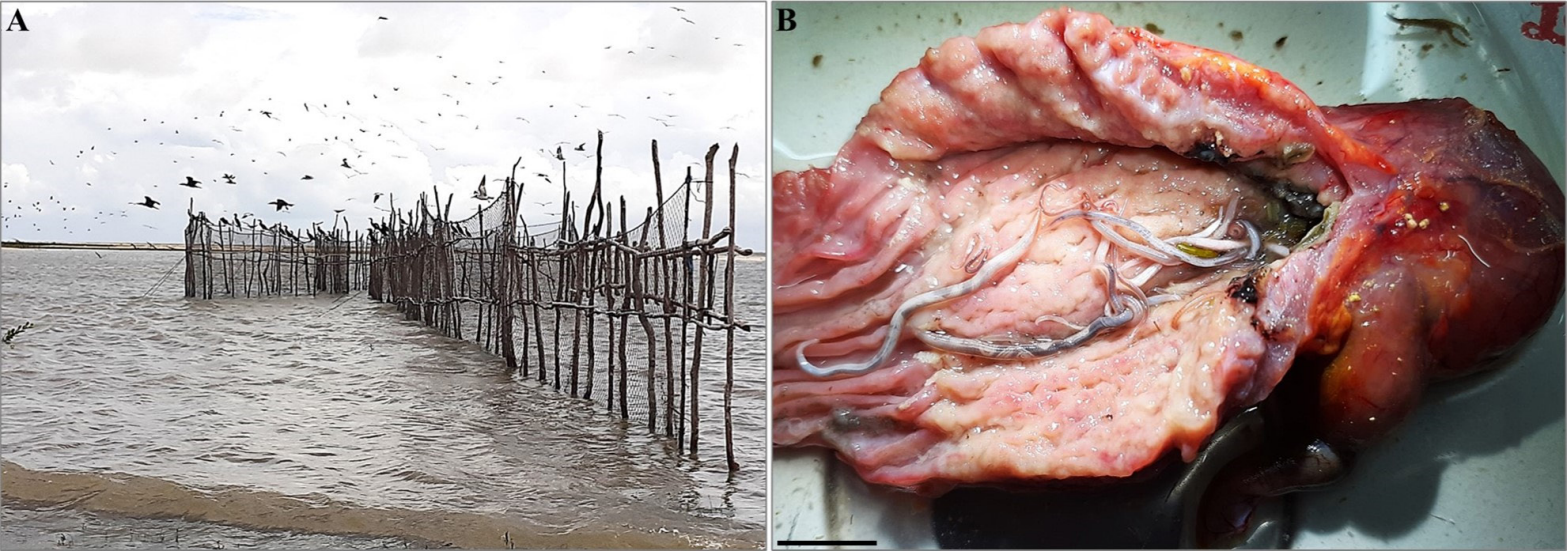
A- Fishing corral located at beach of Mata Fome, Soure Marine Extractive Reserve, the place where the birds were obtained, Marajó Island, State of Pará, Brazil. B- Cormorant stomach containing specimens of *Contracaecum*. (Scale bar: 1 cm).

From 2020 to 2022, twenty specimens of *P*. *brasilianus* were obtained from the coastal zone of the municipality of Soure (Marine Extractive Reserve of Soure) on the Marajó Island, Pará, Brazil (Figure 1) (Latitude: -0.742862°, Longitude: -48.507732°). The birds are used as an alternative source of food by fishers in the region, who kindly provided the dead birds that were used in this study. The animals were transported individually in bags and kept refrigerated in isothermal boxes with ice for transport to the Laboratory. In the laboratory, each organ was individualised in a Petri dish containing 0.9% NaCl saline solution and analysed under a stereomicroscope (Leica ES2), cleaned, quantified, and stored in AFA solution (93 parts of 70% ethyl alcohol, 5 parts of formaldehyde, and 2 parts of glacial acetic acid) for morphological studies, and representative specimens were fixed in 70% ethyl alcohol for molecular analyses.

### Light microscopy

For morphology, the nematodes were clarified in Aman Lactophenol 70%, observed under a microscope (LEICA DM 2500) with a digital capture system (LEICA ICC50 HD) and using the Leica Application Suite software version 4.4.0, being drawn under a microscope (LEICA DM 2500) with attached camera lucida, from which photomicrographs and morphological drawings were respectively obtained. For morphometric analyses, fifteen adult males and fifteen gravid females were used, measuring twenty eggs in each female. Measurements are given in millimetres, unless otherwise indicated, and are presented as mean values followed by minimum and maximum values in parentheses. The taxonomic classification of nematodes was performed according to Baruš et al. (1978), Fagerholm (1991), and Garbin et al. (2011). The ecological indices of parasitism were calculated according to Bush et al. (1997) and Bautista-Hernández et al. (2015).

### Scanning Electron Microscopy

Twelve nematodes (eight males and four females) were washed in distilled water for 1 hour, post-fixed in 1% Osmium Tetroxide (OSO_4_) for 2 hours, and then submitted to dehydration in an increasing series of ethanol from 70% ethanol until 100% for 1 hour in each battery of alcohol, subsequently subjected to the critical point of CO_2_, mounted on metallic aluminium supports (stubs), metallized with gold+palladium, and analysed in a scanning electron microscope (VEGA 3 LMU/TESCAN).

### Molecular and Phylogenetic Analyses

Four adult nematodes (two males and two females) fixed in 70% ethyl alcohol had a fragment of approximately 5 mm from the central region removed after measuring the total length of the specimens for allocation to molecular analyses by sequencing the regions of the first and second internal spacers transcribed from ribosomal DNA (ITS-1, 5.8S, and ITS-2). The rDNA extraction was performed at the Biomolecular Technology Laboratory of the Federal University of Pará, using a DNA extraction kit (Spin Tissue Mini Kit, Stratec®), following the protocol indicated by the manufacturer.

The ITS-1, 5.8S and ITS-2 regions of the rDNA were amplified using primers NC5 (Forward: 5' - GTA GGT GAA CCT GCG GAA GGA TCA TT - 3') and NC2 (Reverse: 5' - TTA GTT TCT TTT CCT CCG CT - 3') (Zhu et al., 1998).

The final reaction volume was 25 μL, with 2.5 μL of reaction buffer (BUFF), 1 μL of MgCl2, 2 μL of dNTP's, 0.5 μL of each primer, 0.2 μL of Taq-DNA polymerase unit, 17.3 μL of H2O, and 1 μL of extracted DNA. The samples were processed in a thermal cycler (Applied Biosystems™ ProFlex™ PCR System, 3 x 32-well) and subjected to the following conditions: 95ºC for 5 minutes followed by 35 cycles at 95ºC for 1 minute (denaturation), 56ºC for 1 minute (annealing), 72ºC for 1 minute (extension), and a final extension at 72ºC for 7 minutes. The amplicons were submitted to 1.5% agarose gel electrophoresis. The PCR product was purified with ExoSAP-ITTM, quantified in Nanodrop equipment, and sequenced using NC5 and NC2 primers in AB 3500 Genetic Analyzer equipment, generating approximately 700 nucleotides each.

For the phylogenetic analyses, eighteen species of *Contracaecum* were included in the inner group, and for the outgroup, two taxa were chosen: *C*. *australe* (ITS-1: HQ389545/ ITS-2: HQ389547), *C*. *chubutensis* (ITS-1: HQ389546/ ITS-2: HQ389548), *C*. *fagerholmi* (ITS-1, 5.8S, ITS-2: JF424599), *C*. *bioccai* (ITS-1, 5.8S, ITS-2: JF424598), *C*. *eudyptulae* (ITS-1: FM177550/ ITS-2: FM177578), *C*. *septentrionale* (ITS-1: AJ634784/ ITS-2: AJ634787), *C*. *variegatum* (ITS-1: FM177531/ ITS-2: FM177541), *C*. *ogmorhini* (ITS-1: FM177542/ ITS-2: FM177549), *C*. *bancrofti* (ITS-1, 5.8S, ITS-2: OP782836), *C*. *overstreeti* (ITS-1, 5.8S, ITS-2: MG515224), *C*. *microcephalum* (ITS-1: FM177524/ ITS-2: FM177528), *C*. *multipapillatum* (ITS-1: AM940056/ ITS-2: AM940060), *C*. *rudolphii* A (ITS-1: JQ071414/ ITS-2: JQ071437), *C*. *rudolphii* B (ITS-1: JQ071412/ ITS-2: JQ071435), *C*. *rudolphii* C (ITS-1, 5.8S, ITS-2: FJ822037), *C*. *rudolphii* D (ITS-1: FM210251/ ITS-2: FM210268), *C*. *rudolphii* E (ITS-1: FM210257/ ITS-2: FM210271), *C*. *rudolphii* F (ITS-1, 5.8S, ITS-2: JF424597) and for the outgroup *Strongylus edentatus* (ITS-1, 5.8S, ITS-2: KP693438) e *S*. *vulgaris* (ITS-1, 5.8S, ITS-2: KP693439).

For phylogenetic reconstruction, alignment was performed with sequences of ribosomal genes available in GenBank having the ITS-1, 5.8S, and ITS-2 regions or the ITS-1 and ITS-2 interval regions using the BioEdit programme (7.2.5). Bayesian inference (BI) analysis were used based on Markov Chain Monte Carlo (MCMC) tree searches performed with MrBayes 3.1.2 (Ronquist & Huelsenbeck, 2003). Two parallel runs of four simultaneous MCMC searches, each with ten million generations, were performed, with a tree being sampled every 500 generations. Results from the first 1000 trees were discarded as burn-in. The remaining trees were analysed in MrBayes to estimate the posterior probability of each node in the phylogenetic reconstruction. The evolutionary model of nucleotide substitution was determined by the Bayesian Information Criterion (BIC) with the JModelTest programme (Posada, 2008), and the most appropriate model chosen was TPM2uf+G. Genetic distances were determined for sequences from the ITS-1, 5.8S, and ITS-2 regions of *Contracaecum* species using PAUP 4.0b (Swofford, 1998).

## Results

### Morphological data

Examination of the specimens revealed morphological characters that resemble descriptions in the literature (Garbin et al., 2011, 2014; Biolé et al., 2012). Below is the morphological characterization. Morphological and morphometric data for *C*. *australe* are presented in Table 1.

**Table 1:** Morphometric comparison between *Contracaecum australe* and *C*. *rudolphii* parasites of Phalacrocoracidae birds in South America.

Characters	*Contracaecum australe*		*Contracaecum australe*		*Contracaecum australe*		*Contracaecum australe*		*Contracaecum rudolphii*	
	Male	Female	Male	Female	Male	Female	Male	Female	Male	Female
Host Localities Site of infection	*Phalacrocorax brasilianus* Pará-Brazil Proventriculus, Ventriculus		*Phalacrocorax brasilianus* Chile Ventriculus		*Phalacrocorax brasilianus* Argentina Ventriculus		*Phalacrocorax gaimardi* Argentina Ventriculus		*Phalacrocorax brasilianus* Brazil Proventriculus, Ventriculus	
Body ^L^	19.71-28.89	22.1−40.94	13.9−28.4	25.44−41.23	19.25−27.37	27−37	15.24−32.33	15.64−36.2	18−31	23−52
Body ^W^	0.43−0.83	0.59−1.09	0.64−0.93	0.66−1.16	0.65−1	0.70−0.90	0.49−0.81	0.65−1.05	0.306−0.598	0.50−1.1
Nerve ring ^a^	0.50−0.69	0.48−0.76	0.58−0.68	0.50−0.68	0.35-0.39	0.4−0.475	0.43−0.60	0.46−0.60	−	−
Deirids ^a^	0.51−0.73	0.52−0.83	0.58−0.79	0.58−0.79	0.35-0.38	0.46−0.55	0.44−0.77	0.49−0.65	−	−
Oesophagus ^L^	2.36−3.64	2.89−5.09	2.62−4.6	1.52−3.95	4.12−4.4	3.62−4.5	2.23−3.45	2.56−3.5	2.4−3.8	2.4−5.4
Intestinal cecum ^L^	1.5−2.66	2.06−3.46	1.56−3.24	1.3−2.86	3.57−4	3.7−4.25	1.6−2.6	1.66−2.57	2.1−2.9	1.6−3.6
Ventriculus ^L^	0.11−0.24	0.18−0.40	0.20−0.38	0.14−0.28	0.1−0.15	0.19−0.23	0.14−0.25	0.2−0.33	−	−
Ventricular appendix ^L^	0.56−1.03	0.56−1.49	0.87−1.41	0.57−0.91	0.75−0.85	0.62−0.92	0.73−1.36	0.69−1.33	0.8−1.2	0.6−1.5
Vulva a	−	7.59−14.15	−	8.25−10.87	−	8.32−8.45	−	4.70−15.36	−	9.7−21.3
Embryonated egg (µm)	−	55−60	−	63−71	−	47−57	−	50−70	−	91−105
Spicule ^L^	9.54−13.91	−	9.6−15.88	−	9.2−10.45	−	7.2−10.44	−	4.5−8.2	−
Precloacal papillae (pairs)	27−38	−	27−32	−	32−40	−	27−43	−	>30^b^	−
Median papilla	1	−	1	−	1	−	−	−	−	−
Postcloacal papillae (pares)	6+1^c^	−	6+1^c^	−	6+1^c^	−	6+1^c^	−	7	−
Tail ^L^	0.15−0.27	0.29−0.49	0.18−0.24	0.28−0.58	0.12−0.35	0.12−0.3	0.17−0.25	0.22−0.40	0.14−0.235	0.20−0.60
Ratio BL/BW	34.81-44.6	37.5-37.6	21.72-30.54*	35.54-38.54*	27.37-29.62*	38.57-41.1*	31.10-39.79*	24.06-34.48*	51.8-58.8*	46-47.27*
Ratio BL/EL	7.94-8.35	7.65-8.04	5.31-6.17*	10.44-16.74*	4.67-6.22*	7.46-8.2*	6.83-9.34*	6.11-10.34*	7.5-8.15*	9.58-9.63*
Ratio BL/TL	107-131,4	76.2-83.55	77.22-118.3*	1.17-1.38*	78.2-160.4*	0.98-1.06*	89.65-128.9*	1.36-1.54*	128.6-131.9*	1.5-1.5*
Ratio EL/ICL	1.37-1.57	1.40-1.47	1.42-1.68*	2.67-4.34*	1.1-1.15*	4.89-5.84*	1.33-1.39*	2.63-3.71*	1.14-1.31*	3.6-4*
Ratio EL/VAL	3.53-4.21	3.42-5.16	3.01-3.26*	71.09-90.86*	5.17-5.49*	123.3-225*	2.54-3.05*	71.09-90.5*	3-3.17*	86.67-115*
Ratio BL/SL	2.07-2.08	-	1.45-1.79^*^	-	2.09-2.62*	-	2.12-3.09*	-	3.78-4*	-
Number of specimens	15	15	10	10	8	4	10	10	30	30
References	Present study		Garbin et al. (2011)		Biolé et al. (2012)		Garbin et al. (2014)		Amato et al. (2006)	

L = length; ^W^= width; BL/BW= body length/body width ratio; BL/EL= body length/oesophagus length ratio; BL/TL= body length/tail length ratio; EL/ICL= oesophagus length/intestinal cecum length ratio; EL/VAL= oesophagus length/ventricular appendix length ratio; BL/SL= body length/spicule length ratio;

a = distance from the anterior region to;

b =more than 30 pairs of precloacal papillae;

c = +1 pair of phasmids;

* ratios calculated with maximum and minimum values provided by the authors.

Ascaridoidea Baird, 1853

Anisakidae Railliet & Henry, 1912

*Contracaecum australe* Garbin, Mattiucci, Paoletti, González-Acuña and Nascetti, 2011

**Figure 2:**
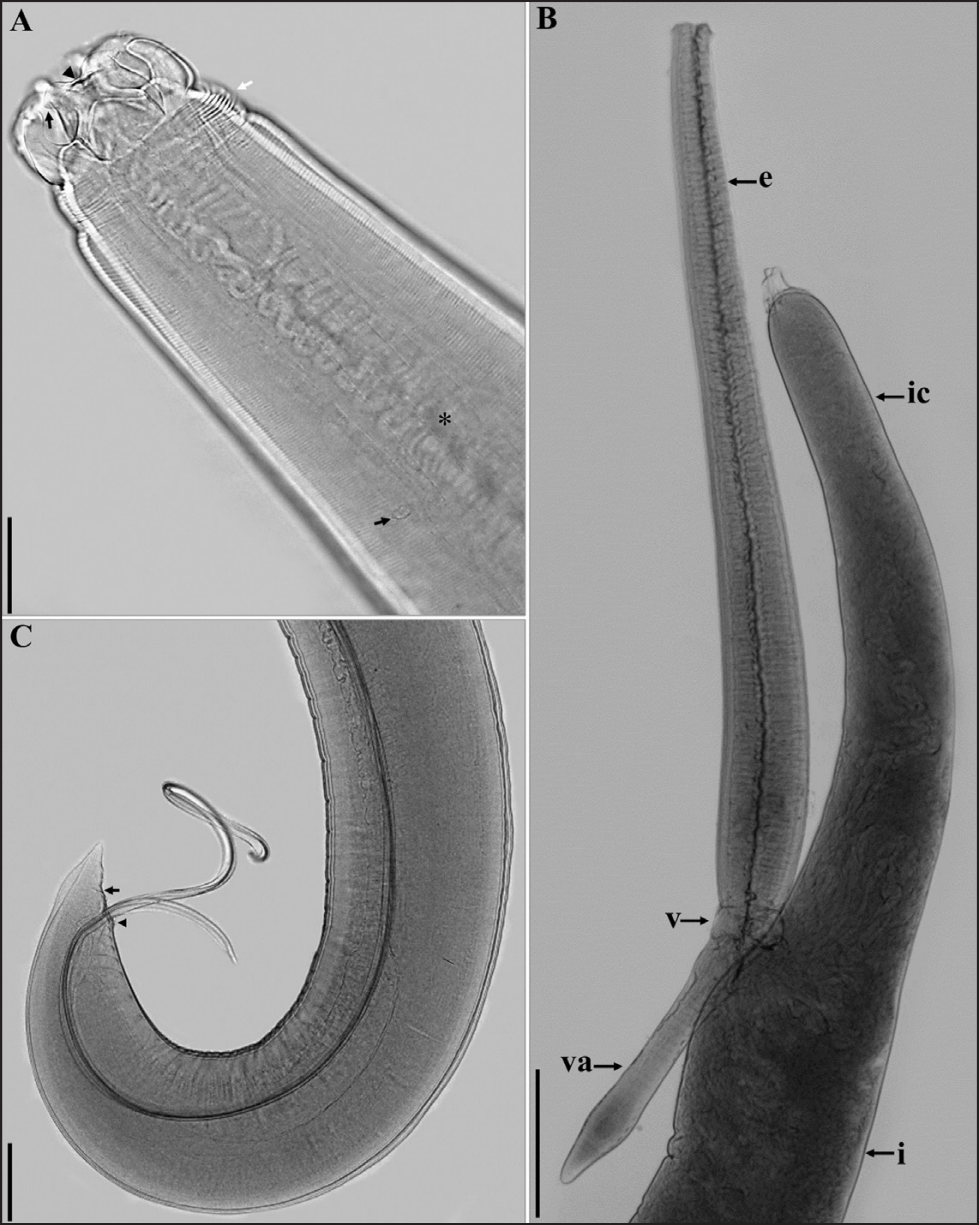
“A-C” Light photomicrographs of *Contracaecum australe*. A- Lateral view of the anterior region where it is possible to observe the ventrolateral lip with a depressed medial apical margin (black head), conspicuous auricle (black arrow), very evident cephalic collar (white arrow), nerve ring (*) and deirids (head of white arrow), scale bar 100 µm. B- Lateral view of part of the dissected digestive system, showing the oesophagus (e), well-developed intestinal cecum (ic), globular ventriculus (v), solid ventricular appendix (va) and intestine (i), scale bar 500 µm. C- Lateral view of the caudal region of a male demonstrating caudal constriction (arrow) and presence of the median papilla on the upper lip of the cloaca (arrowhead), scale bar 300 µm.

**Figure 3:**
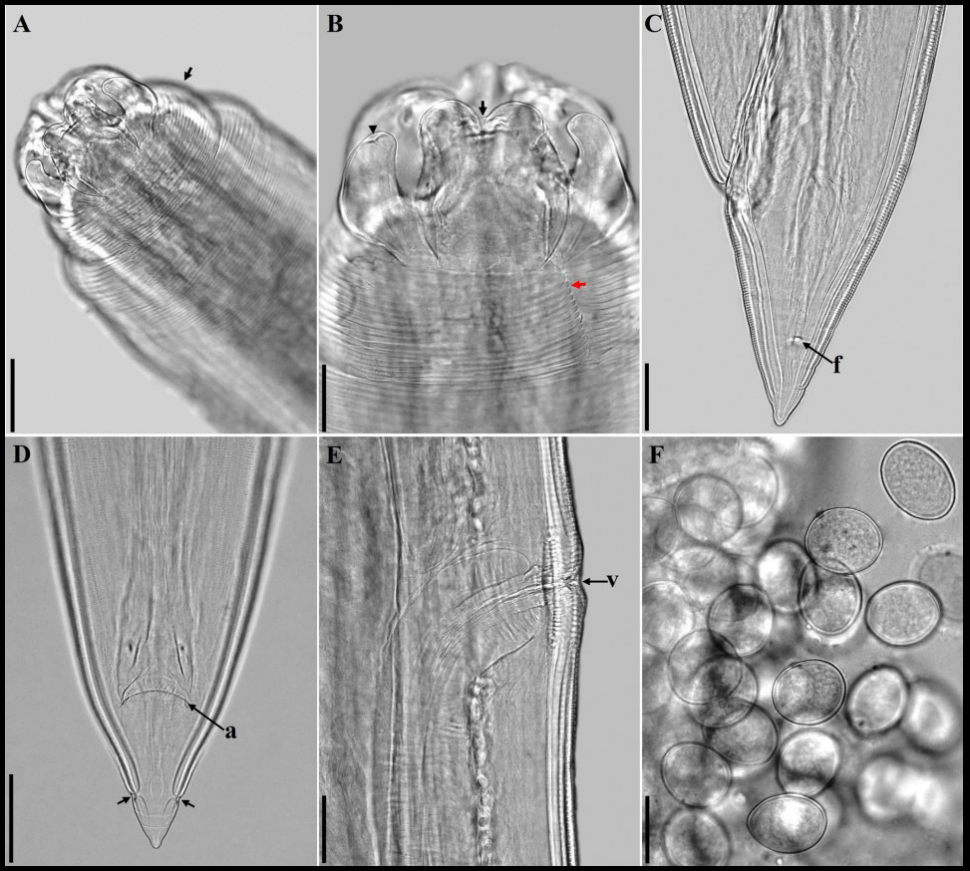
“A-F” Photomicrographs of female from *Contracaecum australe*. In A- Latero-apical view of the cephalic region where interlabia can be observed, conspicuous cephalic collar (black arrow), scale bar 50 µm; B- Lateral view of the anterior region demonstrating the shape of a ventrolateral lip that is longer than it is wide, with a markedly depressed medial apical region (black arrow), interlabia with a bifid apex (arrowhead), cephalic collar with no striations in its lateral region (red arrow), 50 µm scale bar; C- lateral view of the tail of a female showing the phasmid (f), scale bar 100 µm; D- ventral view of the same female tail showing the anus (a) and phasmids (arrows), scale bar 200 µm; E- lateral view demonstrating the shape of the vulva (v), scale bar 100 µm. F- 50 µm scale bar eggs.

**Figure 4:**
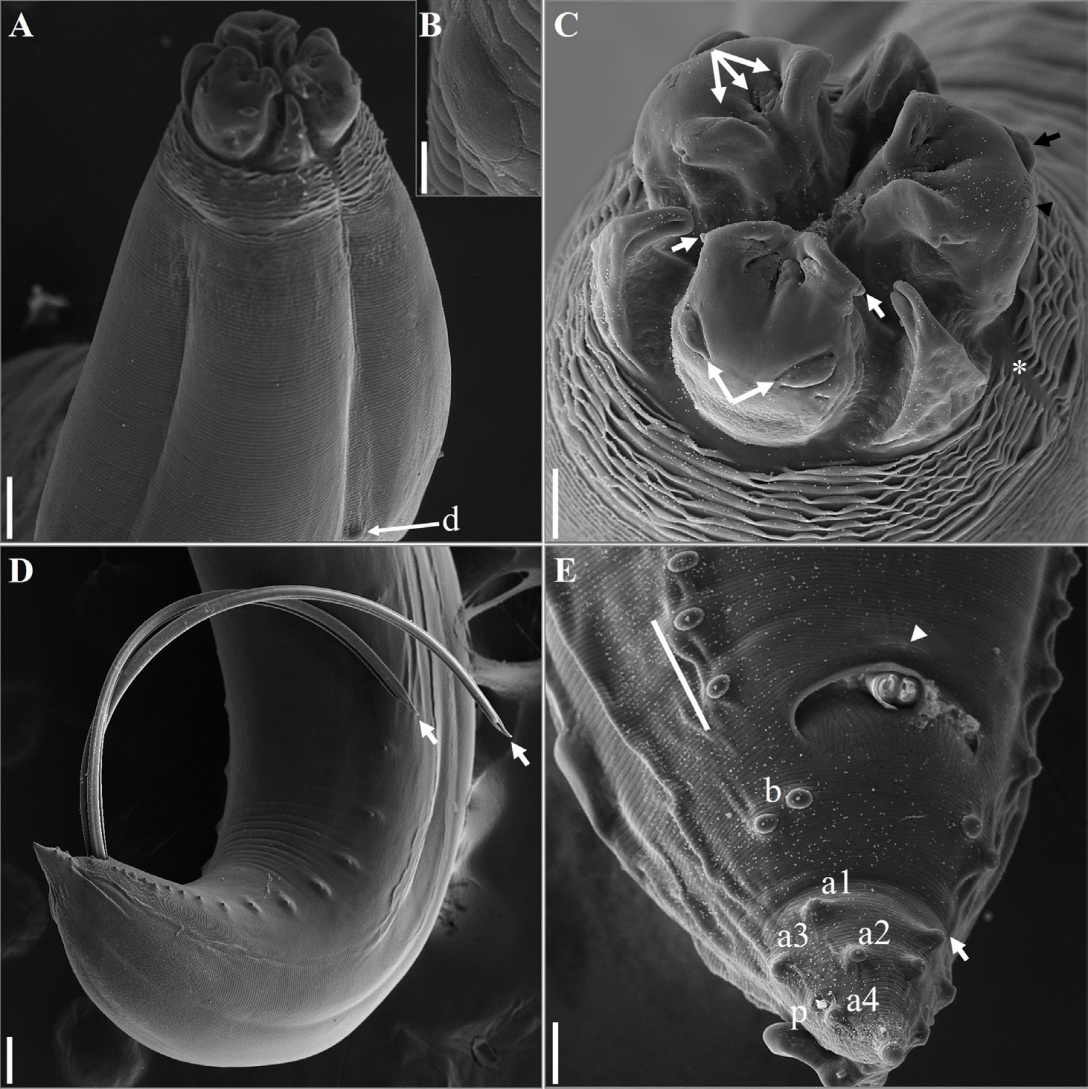
“A-E” Scanning electron microscopy of male *Contracaecum australe*. In A- dorsolateral view showing the presence of the deirids in the form of a button and a smooth surface (d), scale bar 50 µm. B- insert showing the deirid button shape in the largest increase, scale bar 6 µm. C- apical view showing lips with medial apical region with the presence of three clear notches (triple arrow), dorsal lip with the presence of two large papillae (double arrow), and conspicuous auricles (white arrow), interlabia with cleft apex, ventrolateral lips showing the presence of large papilla (black arrow) and amphid (black arrowhead) displaced to the side of the body, well-marked cephalic collar, with absence of striations in the lateral region, V-shaped (*), bar of scale 50 µm. D- lateral view of the tail of a male demonstrating spicules with a rounded free distal tip (white arrows), scale bar 100 µm. E- ventrolateral view of the tail showing the marked caudal constriction (arrow) just after the pairs of paracloacal papillae (b), two pairs of subventral papillae (a1, a2), two pairs of sublateral papillae (a3, a4) and phasmid (p) located between sublateral papillae, presence of median papilla on the upper lip of the cloaca (arrowhead) and pts zone containing 2 papillae, 25 µm scale bar.

**Figure 5:**
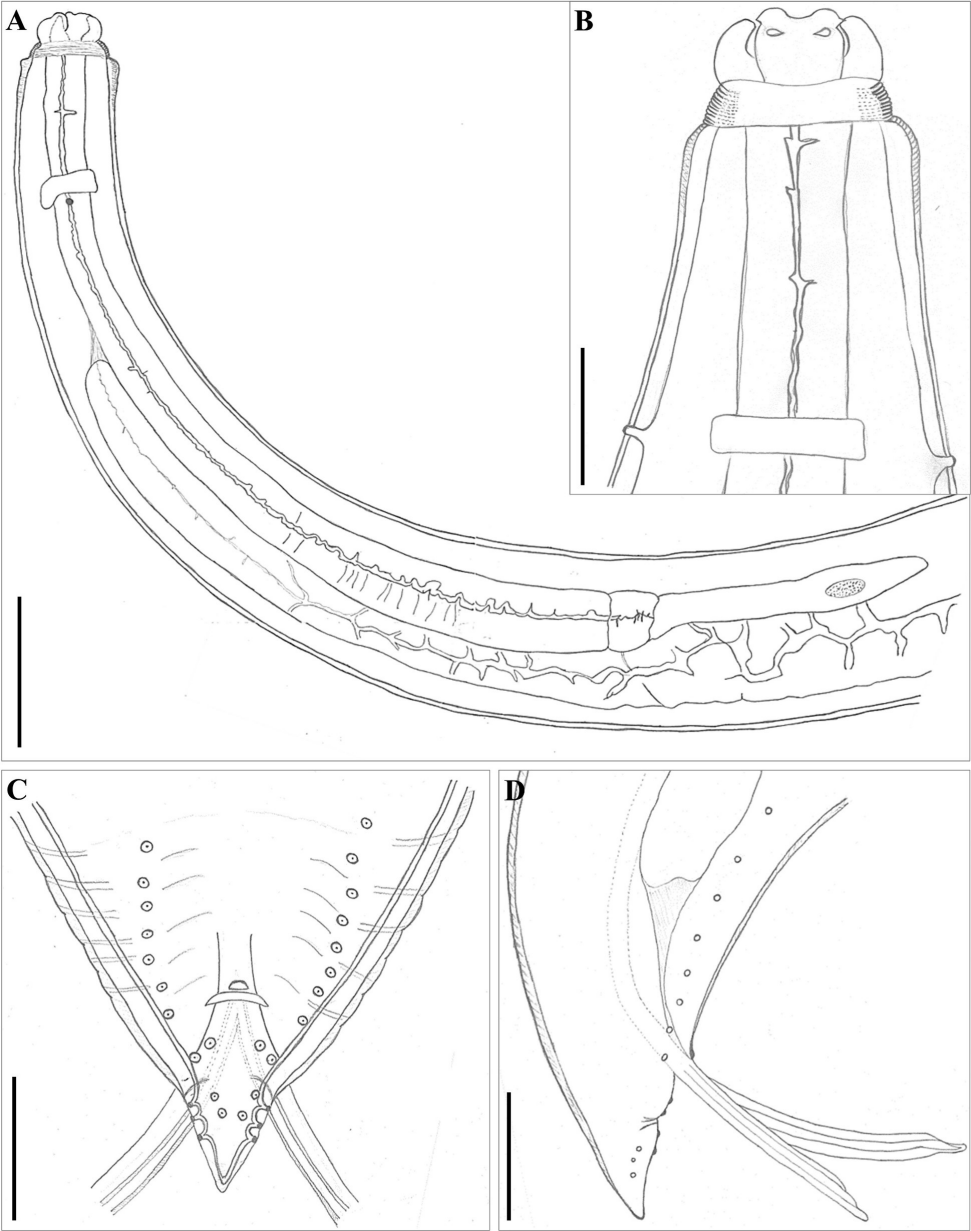
“A-D” Morphological design of *Contracaecum australe*, a parasite of *Phalacrocorax brasilianus* in Northern Brazil. In A- anterior end of body, scale bar 50 µm. B- anterior end, dorsal view, scale bar 20 µm. C- posterior end of male, ventral view, scale bar 20 µm. D- posterior end of male, lateral view, scale bar 20 µm.

Based on light and scanning electron microscopy analyses: (Figures 2-5 and Table 1).

General Morphology (based on 42 specimens): Body totally striated transversely. Very evident cephalic collar with a V-shaped lateral region without striations (Figures 2A, 3A, 3B, 4C, 5A, 5B). Three smooth or slightly cleft interlabia reach 4/5 of the length of the lips. Excretory pore is located immediately below the ventral interlabia. Lips longer than wide, each lip bearing three notable medial apical notches (Figures 3B, 4C), with two conspicuous lobed auricles directed laterally. Dorsal lip with two large laterally directed papillae (Figures 4C, 5B). Ventrolateral lips with one large papilla and a very evident amphid, displaced to the lateral line of the body (Figure 4C). Button-shaped deirids located at the level of the nerve ring or immediately posterior (Figures 2A, 4A, 4B, 5A, 5B). Globular ventriculus, posterior ventricular appendix, developed intestinal cecum, 2 to 3 times longer than ventricular appendix (Figures 2B, 5A). Pre-equatorial vulva. Conical tail. Phasmids clearly visible in both males and females (Figure 3C, 3D).

Males (based on 15 specimens): Mean body length 25.25 (19.71-28.89). Width at the oesophagus-ventriculus junction 0.64 (0.43−0.83). Body length/body width ratio 39.45 (34.81-45.84). Distance from anterior end to nerve ring and deirids 0.56 (0.50−0.69) and 0.69 (0.51−0.73), respectively. Oesophagus length 3.03 (2.36−3.64). Body length/oesophagus length ratio 8.33 (7.94-8.35). Intestinal cecum length 2.1 (1.5−2.66). Oesophagus length/intestinal cecum length ratio 1.44 (1.37-1.57). Ventriculus length 0.2 (0.11−0.24). Ventricular appendix length 0.804 (0.56−1.03). Oesophagus length/ventricular appendix length ratio 3.77 (3.53-4.21). Spicules subequal, reaching almost half the length of the body, measured 12.18 (9.54−13.91), with rounded distal tip. Body length/spicule length ratio (BL/SL): 2.07 (2.07-2.08). Tail length 0.2 (0.15−0.27). Caudal end conical, having 27-38 pairs of precloacal papillae. Pts zone (= first 25 precloacal transverse striae) including 2 pairs of precloacal papillae. Six pairs of postcloacal papillae are present: 2 paracloacal pairs, 2 subventral pairs, 2 sublateral pairs (Figures 4E, 5C, 5D). Body length/tail length ratio 126.2 (107-131.4). A pair of phasmids between the two pairs of sublateral papillae. Median plaque (median papilla) clearly visible on the anterior border of the cloaca (Figures 4E, 5C). Marked caudal constriction just after the paracloacal papillae (Figures 4E, 5D).

Females (based on 15 gravid females and embryonated eggs): Mean body length 29.66 (22.1−40.94). Width at the oesophagus-ventriculus junction 0.78 (0.59−1.09). Body length/body width ratio 38 (37.4-37.5). Distance from anterior end to nerve ring and deirids 0.64 (0.48−0.76) and 0.68 (0.52−0.83), respectively. Oesophagus length 3.98 (2.89−5.09). Body length/oesophagus length ratio 7.45 (7.65-8.04). Intestinal cecum length 2.69 (2.06−3.46). Oesophagus length/intestinal cecum length ratio 1.48 (7.65-8.04). Ventriculus length 0.29 (0.18−0.40). Ventricular appendix length 0.89 (0.56−1.49). Oesophagus length/ventricular appendix length ratio 4.47 (3.42-5.16). Pre-equatorial vulva, found in the first third of the body. Distance from anterior end to vulva 10.64 (7.59−14.15). Tail length 0.37 (0.29−0.49). Body length/tail length ratio 80.2 (76.2-83.55). One pair of distal phasmids (Figure 3C, 3D). Diameter of embryonated egg 58 (55−60µm) (Figure 3F).

## Taxonomic Summary

Host: *Phalacrocorax brasilianus* (Gmelin, 1789) (Aves, Phalacrocoracidae).

Location: Soure Marine Extractive Reserve - Marajó Island, State of Pará-Brazil.

Site of infection: Proventriculus and Ventriculus.

Prevalence: 17 infected out of 20 (85%).

Mean intensity and range: 43.7 (7-360).

### Molecular data

In our study, the tree topology derived from the phylogenetic analyses inferred from the ITS-1, 5.8S, and ITS-2 intergenic regions of the rDNA of the molecularly analysed specimens (GenBank accession number: OQ397677), demonstrated a 100% correspondence with *C*. *australe* (Figure 2), grouping it in the same clade and showing it to be distinct from the other species previously genetically characterized and considered for comparison purposes. Parasitic specimens of *P*. *brasilianus* from Brazil matched previously reported sequences for the ITS-1 and ITS-2 genes of *C*. *australe* characterised in Chile by Garbin et al. (2011) and deposited in GenBank under accession numbers (ITS-1: HQ389545; ITS-2: HQ389547).

A matrix of genetic distances based on the ITS-1, 5.8S, and ITS-2 sequences (2-parameter index from Kimura, 1980) between members grouped according to tree topology is presented in Table 2. The genetic distances between the taxa studied ranged from 0.012 to 0.064. The values between *C*. *rudolphii* C and *C*. *ogmorhini* (0.012) were the lowest observed in this study.

**Table 2:** Genetic distance values inferred from the analysis of ITS-1, 5.8S, and ITS-2 sequences between species of *Contracaecum*.

	(1)	(2)	(3)	(4)	(5)	(6)	(7)	(8)	(9)	(10)	(11)	(12)	(13)	(14)	(15)	(16)	(17)	(18)	(19)	(20)
(1) Study present	-																			
(2) *Contracaecum australe*	0.000	-																		
(3) *Contracaecum chubutensis*	0.021	0.022	-																	
(4) *Contracaecum rudolphii* A	0.046	0.048	0.032	-																
(5) *Contracaecum rudolphii* B	0.032	0.032	0.024	0.018	-															
(6) *Contracaecum rudolphii* C	0.043	0.045	0.029	0.012	0.015	-														
(7) *Contracaecum rudolphii* D	0.044	0.046	0.035	0.022	0.018	0.019	-													
(8) *Contracaecum rudolphii* E	0.046	0.048	0.033	0.018	0.015	0.015	0.016	-												
(9) *Contracaecum rudolphii* F	0.044	0.046	0.033	0.018	0.015	0.015	0.015	0.009	-											
(10) *Contracaecum microcephalum*	0.132	0.132	0.129	0.143	0.125	0.135	0.141	0.135	0.133	-										
(11) *Contracaecum eudyptulae*	0.041	0.043	0.030	0.025	0.020	0.022	0.025	0.025	0.024	0.145	-									
(12) *Contracaecum ogmorhini*	0.043	0.044	0.032	0.016	0.013	0.013	0.013	0.007	0.004	0.131	0.022	-								
(13) *Contracaecum septentrionale*	0.057	0.059	0.044	0.051	0.045	0.048	0.054	0.049	0.046	0.121	0.047	0.048	-							
(14) *Contracaecum fagerholmi*	0.067	0.069	0.065	0.074	0.058	0.071	0.076	0.069	0.067	0.143	0.063	0.069	0.068	-						
(15) *Contracaecum bioccai*	0.059	0.060	0.055	0.067	0.053	0.062	0.072	0.064	0.063	0.145	0.059	0.062	0.061	0.044	-					
(16) *Contracaecum overstreeti*	0.563	0.562	0.549	0.542	0.555	0.552	0.555	0.557	0.564	0.538	0.555	0.560	0.559	0.593	0.570	-				
(17) *Contracaecum bancrofti*	0.248	0.250	0.246	0.258	0.237	0.253	0.264	0.249	0.256	0.247	0.254	0.249	0.249	0.272	0.257	0.491	-			
(18) *Contracaecum multipapillatum*	0.526	0.525	0.513	0.504	0.518	0.513	0.515	0.517	0.524	0.498	0.518	0.521	0.515	0.547	0.525	0.051	0.455	-		
(19) *Contracaecum variegatum*	0.046	0.048	0.033	0.019	0.013	0.013	0.021	0.016	0.016	0.129	0.024	0.015	0.051	0.067	0.060	0.561	0.259	0.523	-	
(20) *Strongylus edentatus*	200.929	201.366	202.207	199.080	201.449	200.181	195.991	202.939	201.390	244.617	197.807	202.597	210.689	210.485	208.835	212.943	191.009	211.865	202.826	-
(21) *Strongylus vulgaris*	198.510	198.962	198.694	203.889	204.518	202.794	199.211	204.572	203.778	240.223	197.803	204.213	208.147	215.524	212.098	210.092	192.109	203.905	203.002	0.198

## Discussion

In this study, nematodes recovered from the proventriculus and ventriculus of *P*. *brasilianus* on the north coast of the State of Pará, presented morphological characters compatible with *C*. *australe* (Garbin et al., 2011; 2014; Biolé et al., 2012), making it possible to assign them to this specific taxon, which was found parasitizing *P*. *brasilianus* from the Marine Extractive Reserve of Soure, Pará-Brazil.

Among the *Contracaecum* species that occur in Brazil, this is the first record of *C*. *australe* in the national territory. This species was described by Garbin et al. (2011) in the Santa Elena lagoon in Chile as a parasite of *P*. *brasilianus* using morphological and molecular techniques. Biolé et al. (2012) recorded the species on the same host in the central Argentina region, and later *P*. *gaimardi* was added as a new host for *C*. *australe* in the southernmost record of the species in Argentina, thus expanding its geographical distribution and definitive host range (Garbin et al., 2014).

Garbin et al. (2011), when describing *C*. *australe* based on morphological characters considered diagnostic for the species of the genus (*sensu* Hartwich, 1964), such as the length of the spicules, morphology of the distal end of the spicule, and the presence of a slit in the interlabial tip, reported that, a priori, this parasite species of *P*. *brasilianus* from Chile could be easily attributed to *C*. *rudolphii lato sensu*. However, after the morphological comparison of the new specimens and due to the presence of characters such as a well-marked constriction in the tail just after the pairs of paracloacal papillae, presence of a median plaque (median papilla), parasites being apparently smaller, more robust and presenting longer spicules than *C*. *rudolphii* s.l., and supported by phylogenetic analyses of sequences from multiple loci, it was confirmed as a highly supported clade distinct from the rest of the *Contracaecum* taxa considered, thus validating its specific status.

Observing the morphological characteristics of *C*. *australe* and comparing them to their parasitic congeners of birds, we can see that this species can be easily distinguished from several of them using morphological characters with high diagnostic value (Fagerholm 1988, 1991; Moravec & Scholz, 2016), such as *C*. *multipapillatum* s.l.; *C*. *pyripapillatum*; *C*. *overstreeti*; *C*. *gibsoni*; *C*. *bancrofti*; *C*. *spasskii*; *C*. *tricuspis*; *C*. *mexicanum*; *C*. *ovale*; *C*. *heardi*; *C*. *variegatum*, *C*. *travassosi* that have one or more pairs of double postcloacal caudal papillae. However, sometimes this morphological differentiation is more difficult and requires the use of distinctive diagnostic characters together, such as body length/body width ratio, body length/spike length ratio, and oesophagus length/ventricular appendage length ratio, among others. Garbin et al. (2011, 2014), to differentiate between species, such as between *C*. *australe* and *C*. *rudolphii* complex, as these species have a large similarity in the number and pattern of distribution of caudal papillae.

According to Moravec & Scholz (2016), the shape and length of cephalic structures, such as lips and interlabia, the number and arrangement of pre- and postcloacal papillae, as well as the shape of the distal end of the spicules, are taxonomic features that distinguish between *C*. *rudolphii* and three other congeners parasites of birds, *C*. *microcephalum*, *C*. *micropapillatum,* and *C*. *variegatum*. Currently, the *C*. *rudolphii* complex has six described cryptic species (*C*. *rudolphii* A, B, C, D, E, and F) (D'Amelio et al., 2007, 2012; Mattiucci et al., 2008; Shamsi et al., 2009b).

*Contracaecum australe* can be differentiated from *C*. *fagerholmi* and *C*. *rudolphii* F described by D'Amelio et al. (2012), by the length of the spicules that vary from (4.15-4.85 and 5.96-7.30 mm), respectively, as opposed to (9.6-15.88 mm) in *C*. *australe* described by Garbin et al. (2011), values like those found in the present study (9.54-13.91 mm). However, Biolé et al. (2012), when noting the occurrence of *C*. *australe* in *P*. *brasilianus* in Argentina, saw morphometric variations that were considered intraspecific variations, until molecular studies can prove otherwise or corroborate their results. The authors observed a more anterior position of the nerve ring and deirids, a smaller ventriculus and ventricular appendix, a greater number of precloacal papillae, pre-equatorial location of the vulva, and smaller size of the eggs.

Garbin et al. (2014), when adding a new host parasitized by *C*. *australe* also in Argentina (*P*. *gaimardi*), pointed out morphometric variations that partially corroborated the findings of Biolé et al. (2012), such as greater amplitude in the number of precloacal papillae, smaller size of the ventriculus and ventricular appendix in male specimens, more pre-equatorial vulva and smaller size of eggs in females. However, the most important morphometric difference occurred in the length of the spicules (7.2-10.44 mm), which were almost a third shorter than those described in specimens found in *P*. *brasilianus* from Chile (9.6-15.88 mm) and in the present study (9.54-13.91 mm). And as we can observe within the congener species of *Contracaecum* parasites of piscivorous birds, there is a wide range of variation in several metric characters that allow the fitting of multiple species and make it difficult to clearly differentiate.

*Contracaecum australe* can be differentiated from *C*. *jorgei,* also recorded in *P*. *brasilianus* (Syn. *Nannopterum brasilianus),* by the greater length of the spicules (9.54−13.91 vs 2.03-3.63) and the greater number of precloacal papillae (27-38 vs 26), respectively. Furthermore, when describing the species, Sardella et al. (2020) reported the presence of two papillae on the ventrolateral lips, as well as the dorsal lip being longer than the ventrolateral lips, whereas in *C*. *australe* the lips are the same size and the ventrolateral lips have only one labial papilla and a phasmid in each (Garbin et al., 2011). These results are similar to those found in the present study for the species.

According to Garbin et al. (2011), morphological analyses and differential diagnosis of male specimens of *C*. *australe* allowed the detection of differences in several characters, including the length of the spicule, the peculiar shape of the male tail, the disposition of the paracloacal papillae, and the depth and shape of the cleft in the interlabium. As for the characters mentioned by the authors, such as caudal constriction after the paracloacal papillae and presence of the median plaque (median papilla), they have not proven to be strong characters for differentiating, for example, between *C*. *australe* and *C*. *rudolphii* s.l., since, despite not having been described by some authors, they seem to be clearly present in their illustrations or even photomicrographs. (See, for example, Abollo et al., 2001; Amato et al., 2006; D'Amelio et al., 2012; Moravec & Scholz, 2016), as previously stated by the authors (Garbin et al., 2011).

Garbin et al. (2014) suggested that the specimens described by Amato et al. (2006) as *C*. *rudolphii* parasites of *P*. *brasilianus* in Brazil may be *C*. *australe*, because they share certain morphological characteristics (such as lips, interlips, arrangement, and number of caudal papillae) and the same host. However, when taking into account the length of the spicules and the BL/SL ratio between (*C*. *rudolphii* 4.5-8.2 mm and 3.8-4 Amato et al., 2006) vs (9.6-15.88 mm and 1.45-1.79 *C*. *australe*Garbin et al., 2011) and (9.54−13.91 mm and 2.07-2.08 *C*. *australe* present study), we can see that these species clearly differ and show that these morphological characters seem to be the most consistent ones for differentiating between species, given that, so far, the species of the *C*. *rudolphii* complex described molecularly and morphologically have smaller spicules than *C*. *australe.* *C*. *rudolphii* D 3.90-6.99 mm; *C*. *rudolphii* E 5.53-6.13 mm Shamsi et al. (2009b); *C*. *rudolphii* F 5.96-7.3 mm D’Amelio et al. (2012), and higher BL/SL ratios in the species of the *C*. *rudolphii* complex (*C*. *rudolphii* D 3.7-3.8; *C*. *rudolphii* E 4.3-4.4; *C*. *rudolphii* F 2.5-2.7) than in *C*. *australe* (1.45-1.79 Garbin et al. 2011; 2.07-2.08 present study). The sister species *C*. *rudolphii* A, B, and C, despite having been characterised molecularly, have not been morphologically described, and only the length of the spicules is available for these species (D'Amelio et al., 2007; Mattiucci et al., 2008).

We agree with Garbin et al. (2014) when they state the need to review the specimens described by Amato et al. (2006) and, if possible, evaluate them molecularly to complement the morphological diagnosis of these parasites. As previously seen, *P*. *brasilianus* is a bird capable of harbouring multiple species of *Contracaecum* at the same time (Lent & Freitas, 1948), raising the possibility that this bird presents co-infection with *C*. *australe* and *C*. *rudolphii* s.l., and/or more species of the genus in the same individual (Ricardo et al., unpublished data).

For Sardella et al. (2020), the use of molecular techniques is fundamental not only for defining the taxonomic status of these species but also for enabling their recognition. Result observed in the topology of the tree, derived from the inferred phylogenetic analysis of the ITS-1, 5.8S and ITS-2 genic regions of the rDNA of four specimens analysed molecularly in our study, being observed 100% of correspondence with *C*. *australe* described in Chile (Garbin et al., 2011), if grouping in the same corresponding clade the previously reported sequences for the ITS-1 and ITS-2 genes deposited in GenBank.

**Figure 6:**
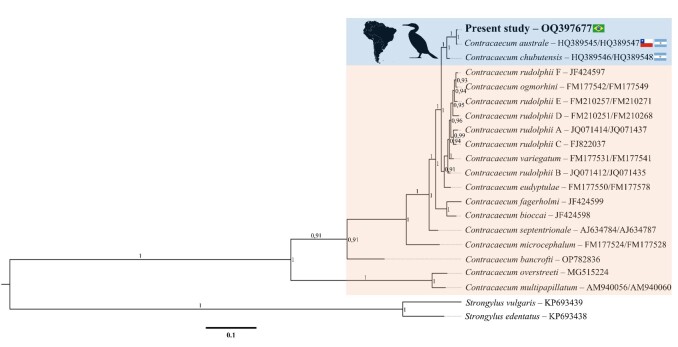
Phylogenetic tree inferred by Bayesian analysis (BI) of *Contracaecum* species, based of Internal Transcribed Spacer ribosomal gene sequences (ITS-1, 5.8S, and ITS-2) and using *Strongylus edentatus* and *Stongylus vulgaris* as outgroups. Node numbers represent posterior probability values recovered by the Bayesian analysis.

In our study, the clade formed in the phylogenetic tree by *C*. *australe* specimens was distinct from all *Contracaecum* species previously genetically characterized and considered for comparison purposes. In the phylogenetic analyses, it was possible to observe that *C*. *chubutensis* was the species that was genetically closest to *C*. *australe*, with a distance of 0.021, but forming two distinct clades. This genetic proximity can be justified by both species being parasites of birds (Phalacrocoracidae) and having the same biogeographical distribution (See Figure 6). However, the smallest genetic distance seen in our study occurred between the species *C*. *rudolphii* F and *C*. *ogmorhini* (0.004). Small genetic distances were also observed between *C*. *ogmorhini* and *C*. *rudolphii* E (0.007) and between *C*. *rudolphii* E and *C*. *rudolphii* F (0.009). Furthermore, the analyses of the data from the ITS-1, 5.8S, and ITS-2 sequences of *C*. *australe* from Brazil supported its distinction from the cryptic species of the *C*. *rudolphii complex*, corroborating the results found by Garbin et al. (2011).

## Conclusion

*Phalacrocorax brasilianus* from the north coast of Brazil is the definitive host of *C*. *australe*; this is the first record of the species in the national territory. In this study, we have expanded the biogeographical distribution of this parasite, in addition to highlighting the need for the application of integrative taxonomy for the characterization of species of *Contracaecum*.
